# Auditory Perceptual History Is Propagated through Alpha Oscillations

**DOI:** 10.1016/j.cub.2019.10.041

**Published:** 2019-12-16

**Authors:** Hao Tam Ho, David C. Burr, David Alais, Maria Concetta Morrone

**Affiliations:** 1 School of Psychology, University of Sydney, Brennan MacCallum Building A18, Manning Road, Camperdown, NSW 2006, Australia; 2 Department of Translational Research on New Technologies in Medicine and Surgery, University of Pisa, Via San Zeno 31, 56123 Pisa, Italy; 3 Department of Neuroscience, Psychology, Pharmacology, and Child Health, University of Florence, Via di San Salvi 12, 50139 Florence, Italy; 4 Institute of Neuroscience, Via Giuseppe Moruzzi 1, 56124 Pisa, Italy

**Keywords:** audition, serial dependence, perceptual history, alpha rhythm, behavioral oscillations, working memory, signal detection theory, decision criterion, response bias, perceptual echo

## Abstract

Perception is a proactive, “predictive” process, in which the brain relies, at least in part, on accumulated experience to make best guesses about the world to test against sensory data, updating the guesses as new experience is acquired. Using novel behavioral methods, the present study demonstrates the role of alpha rhythms in communicating past perceptual experience. Participants were required to discriminate the ear of origin of brief sinusoidal tones that were presented monaurally at random times within a burst of uncorrelated dichotic white noise masks. Performance was not constant but varied with delay after noise onset in an oscillatory manner at about 9 Hz (alpha rhythm). Importantly, oscillations occurred only for trials preceded by a target tone to the same ear, either on the previous trial or two trials back. These results suggest that communication of perceptual history generates neural oscillations within specific perceptual circuits, strongly implicating behavioral oscillations in predictive perception and with formation of working memory.

## Highlights

• We demonstrate the role of alpha rhythms in the propagation of perceptual history
• Auditory decisions were *rhythmically* biased by stimuli presented 1 or 2 trials back
• Bias oscillated at ∼9 Hz only when successive stimuli occurred in the same ear
• Alpha is strongly implicated in predictive perception and working memory formation

## Graphical Abstract

**Figure:**
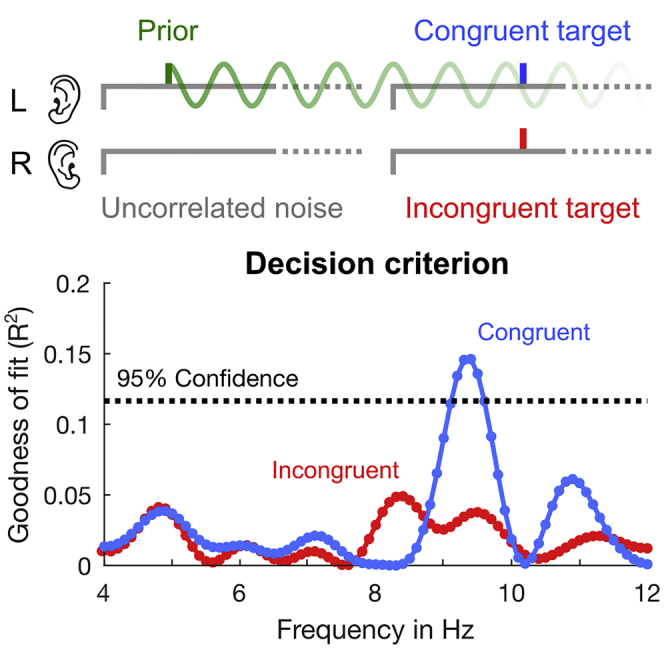

## Teaser

Using novel behavioral methods, Ho et al. show that perceptual decisions in audition are *rhythmically* biased by previous stimuli presented up to two trials back. The oscillations at ∼9 Hz occurred only when a stimulus had previously been presented to the same ear, strongly implicating alpha in predictive perception and working memory formation.

## Introduction

It has long been known that perception depends heavily on expectations and perceptual experience. Helmholtz [1] introduced the concept of “unconscious inference,” suggesting that perception is at least partly “inferential” or “generative,” and Gregory [2] described perception as a series of hypotheses to be verified against sensory data, using many compelling illusions to support this notion. In this view, perception is a proactive, “predictive” process, where the brain uses accumulated experience to make best guesses about the world to test against sensory data, updating the guesses as new experiences are acquired.

Recent studies using “serial dependence” demonstrate clearly the action of predictive perception and provide a means of quantitative study: under many conditions, the appearance of images in a sequence depends strongly on the stimulus presented just prior to the current one. Judgments of orientation [3], numerosity [4], motion [5], facial identity or gender [6, 7], beauty, and even perceived body size [8] are strongly biased toward the previous image. Serial biases are also observed in audition for pitch discrimination [9, 10]. Sequential effects can last up to minutes [11], showing that perception does not rely solely on instantaneous stimulation but also on predictions, or “priors,” conditioned by events over a long time course.

The neuronal mechanisms underlying serial dependence are largely unknown. It is assumed that the predictions are generated at mid–high levels of analysis and fed back to early sensory areas, whose activity in turn feeds forward to add to the accumulated knowledge and shape future predictions [12, 13, 14, 15, 16]. Little is known, however, about how this information is propagated, or the nature of the underlying neural mechanisms. One possibility is that recursive propagation and updating of stored prior experience are related to low-frequency neural oscillations [17, 18, 19].

We have recently shown, in both audition [20] and vision [21], that “sensitivity” (accuracy) and “criterion” (response bias) are not constant but oscillate rhythmically over time at different frequencies: theta for sensitivity and alpha for criterion, suggesting separate mechanisms [20, 21]. The oscillations in audition were revealed using monaural stimuli, which may explain the discrepant results of previous studies [22, 23, 24, 25] that used binaural stimuli, potentially generating oscillations out of phase in each ear (discussed in [20]). The alpha oscillations in criteria are consistent with an increasing number of electroencephalography (EEG) findings showing an association between criterion shifts, memory, and modulations of alpha power and phase [18, 26, 27, 28, 29, 30].

Oscillations in bias could plausibly reflect the action of predictive mechanisms, possibly via reverberation of recursive error propagation within a generative framework. Storage of prior information necessarily implicates memory processes. VanRullen and Macdonald [31] have proposed an oscillatory mechanism by which past perceptual visual history may be stored in short-term memory as a reverberatory “perceptual echo.” The reverberation should affect the predictive mechanism that biases perceptual decisions, giving rise to sequential effects. Given the oscillatory nature of the reverberation, behavioral oscillations in criteria, observed in different domains and tasks [20, 21, 32], may be modulated or even gated by the history of the previous presented stimuli. It is still debated as to whether a form of perceptual echo may exist in audition [33], but we test here for this possibility by measuring behavioral oscillations in criterion in a dichotic auditory discrimination task, analyzing the series based on the congruency of previous auditory stimuli. The results demonstrate that perceptual oscillations occur only for stimuli that are congruent with previous stimuli, consistent with an auditory perceptual echo within sensory channels.

## Results

### Average Effects of Stimulus History

**Figure 1:**
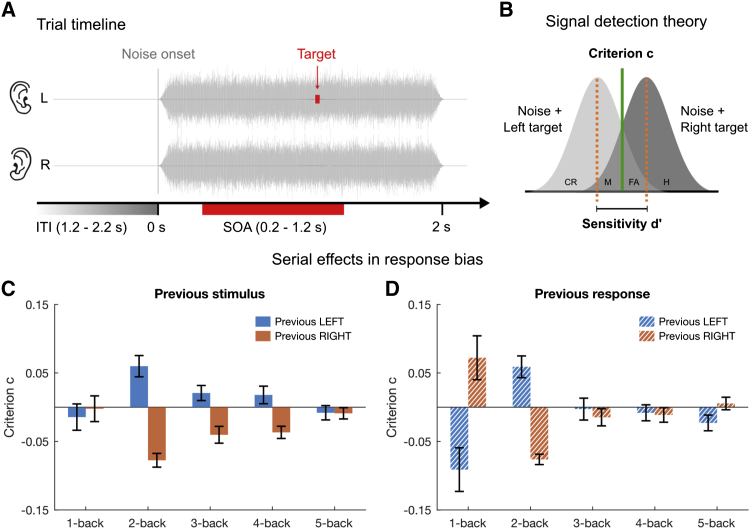
Experimental Design and Results of the Serial-Dependence Analysis (A) Schematics of a trial. On each trial, uncorrelated dichotic white noise was presented simultaneously for 2 s. A pure tone of 1 kHz and 10-ms duration was delivered with equal probability to the left or right ear, at an SOA randomly selected from an interval of 0.2 s to 1.2 s post noise onset. The inter-trial interval varied randomly between 1.2 s and 2.2 s. (B) Application of signal detection theory (SDT). We calculated sensitivity (d′) and decision criterion (c) using the hits (H) and false alarms (FA) from the left- and right-target conditions, respectively (M, misses; CR, correct rejections). The calculations follow Equations 1 and 2. (C) Results of the overall serial-dependence analysis. Group mean response bias (as measured by the decision criterion c; Equation 2) contingent on the ear of origin of the preceding 1–5 stimuli. The difference between the contingent left and right (blue and red bars, respectively) are significant for the 2-, 3-, and 4-back stimuli (Bonferroni corrected). Error bars indicate ± 1 SEM. (D) Group mean response bias contingent on the response 1–5 trials back. The difference between the contingent left and right (blue and red bars, respectively) is significant for 2-back and marginally significant for 1-back (FDR corrected). d′ (Figure S1) showed no significant sequential effects for either 1- or 2-back trials after Bonferroni correction. Error bars indiciate ± SEM.

Participants were required to identify in two-alternative forced choice (2AFC) the ear of origin of a brief monaural near-threshold tone embedded within a 2-s burst of dichotic white noise (Figure 1A). Given the many studies that have shown how stimulus history can bias responses [3, 4, 6, 34, 35, 36], we first looked for average effects in response bias, as measured by criterion (Equation 2; see also Figure 1B). Figure 1C reports the effects of previous trials as a function of relative position in the sequence. Although there was no significant 1-back effect (Bonferroni-corrected p = 3.69, log Bayes factor [logBF] = −0.55), there was a strong and highly significant 2-back effect of response biases toward the previously presented stimulus (p = 0.0002, logBF = 2.91). The effects remained significant for stimuli three and four trials back (p = 0.03, logBF = 0.93 and p = 0.04, logBF = 0.84, respectively), but were not significant five trials back (p = 4.76, logBF = −0.57).

Because it appeared strange to have a 2-back but not a 1-back effect on criterion, we further analyzed the data for a dependency of the *previous response*, to see whether this would help to explain the paradoxical result. Figure 1D shows a *negative* influence of one trial back, only marginally significant after Bonferroni correction (p = 0.1, logBF = 0.49). Although not statistically robust, this repulsive effect could result from the known effect of “response switching” [37], which could explain the lack of stimulus-based serial dependence in 1-back trials. Response dependence on two trials back (which should be enhanced by double-response switching) showed the same assimilative aftereffect found for the stimulus-based analysis (p = 0.0001, Bonferroni corrected, logBF = 3.16). Trials further in the past showed no significant effects (all p values > 0.05).

We also looked for serial-dependence effects in sensitivity (d′; Equation 1; see also Figure 1B). As may be expected (given that the order of trials was completely random), stimulus history had no significant effect on observer sensitivity (Bonferroni-corrected p = 0.06 with logBF = 0.66 and p = 2.36 with logBF = −0.47, respectively, for 1- and 2-back trials; see Figure S1).

### Oscillation of Response Bias, but Not Sensitivity

**Figure 2:**
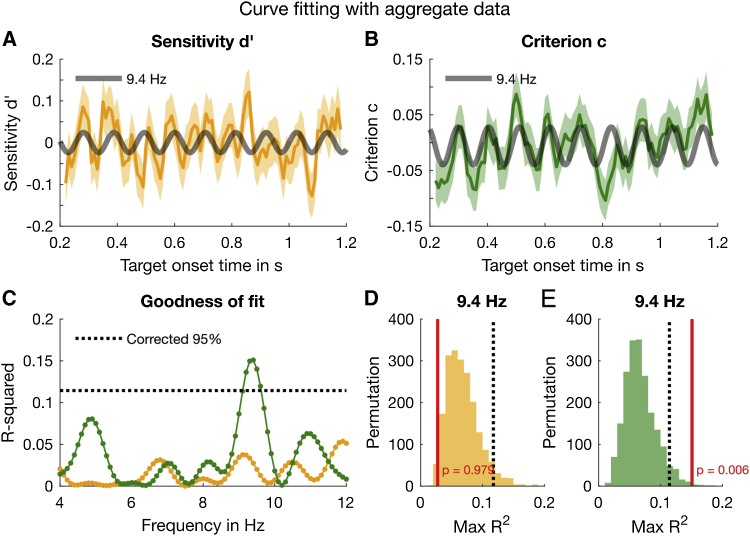
Results of the Curve-Fitting Analysis (A) The yellow line shows the time course of the detrended d′ based on the aggregate data. The data are smoothed for display purposes, with all statistical analyses run on *non*-smoothed data. The shaded yellow area enveloping the line represents bootstrapped ±1 SEM. The gray curve depicts a 9.4-Hz oscillation fitted to the sensitivity data. (B) Same as (A) for criterion (green line). (C) The goodness of fit (R^2^) for all sinusoids from 4 to 12 Hz, in steps of 0.1 Hz. The dotted black line represents the 95^th^ percentiles of the permutation distribution depicted in (E). R^2^ for sensitivity (yellow line) never reaches significance, whereas that for criterion does for the range of 9.1–9.6 Hz, highest at 9.4 Hz. (D and E) Illustration of the permutation method: we shuffled the aggregate data 2,000 times, fitted the shuffled data with the best frequency over the 4- to 12-Hz range, and calculated the distributions of R^2^ for sensitivity (D) and criterion (E) distributions. The red lines show the R^2^ of the fit to the original data at 9.4 Hz. The p value of the sign test is given by the proportion of permuted R^2^ greater than the original R^2^ (red lines). An overview of the statistical results is provided in Table S1.

Figures 2A and 2B show the variation over time in sensitivity (Figure 2A) and criterion (Figure 2B), computed by binning aggregate data as a function of target stimulus-onset asynchrony (SOA) from noise onset. It is evident that criterion oscillates strongly and regularly and can be well fit by a pure sinusoidal function with a frequency of 9.4 Hz, shown by the gray curve in Figure 2B. In contrast, sensitivity does not show a rhythmic periodicity and no sinusoidal function fit the temporal series well (Figure 2C). However, the reader is referred to the post hoc analysis in Figure 5, which shows that when considering the ears separately (not possible for the analysis of sensitivity in this study), accuracy does oscillate, out of phase in each ear.

To evaluate the goodness of fit of each sinusoidal function, we compared the explained variance (R^2^) of the real data for every frequency from 4 to 12 Hz in steps of 0.1 Hz with the R^2^ of the best fit of the surrogate shuffled data at any frequency within the range, to correct for multiple comparisons (Figures 2C–2E; STAR Methods). For sensitivity (orange line), no frequency produced a fit near the 95% confidence threshold (dotted line), corrected for multiple comparisons across all tested frequencies. However, the criterion data (green line) showed significant modulations between 9.2 and 9.6 Hz, with a strong peak at 9.4 Hz (R^2^ = 0.15). The phase and amplitude of the 9.4-Hz oscillation at trial onset relative to the noise burst onset were 179° ± 18° SEM and 0.039 ± 0.011 SEM (by bootstrap), respectively. For sensitivity (left), the explained variance is clearly not significant, whereas for criterion (right) the best fit is higher than 99.1% of the surrogate best fits, giving a sign-test significance (on the goodness of fit) of p = 0.006 (corrected across all tested frequencies). In addition, we evaluated the amplitude and phase of the aggregate fit using a two-dimensional (2D) bootstrap test (as in Figure 7 and similar to the 2D sign test in our previous study [20]). The results, p = 0.01 (corrected with false discovery rate [FDR] = 0.05 [38]), corroborate the sign test of the goodness of fit. Tables S1 and S2 provide an overview of the statistical results in the aggregate and individual data analyses.

**Figure 3:**
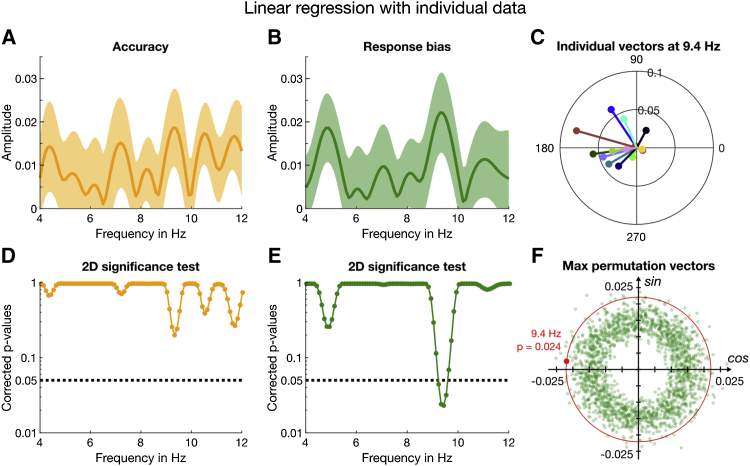
Results of the Linear Regression Analysis Based on Individual Data without Binning (A) The yellow line represents the amplitude spectrum for accuracy computed from the vectorial average of the GLM estimates of β_1_ and β_2_ across participants. The shaded area around the line indicates ±1 SEM. (B) Amplitude spectrum of response bias based on the same analyses as for accuracy. (C) Individual 2D vectors (β_1_, β_2_) at 9.4 Hz for response bias. The length and direction of the line indicate the amplitude and phase (relative to time of noise onset). (D) The results of the 2D permutation test for accuracy for the frequency range of interest, 4–12 Hz, corrected for multiple comparisons. (E) Corrected p values for response bias obtained by the same 2D permutation as for accuracy. (F) Illustration of the 2D permutation test by which the p values in (D) and (E) were computed. The sign test is based on the proportion of the largest permutation vectors (irrespective of frequency) whose amplitudes exceed the group mean (outside the red circle passing through the group mean, shown by the red dot). The statistical results are also summarized in Table S2.

The results shown in Figure 2 were based on an aggregate data analysis, pooling all trials across subjects and fitting sinusoids over the entire duration. In a complementary analysis, we evaluated the consistency across subjects using a general linear model (GLM) approach on single trials that is more resilient to sparse sampling than curve fitting (see also [32, 39, 40]). Specifically, we applied the linear regression in Equation 6 (STAR Methods) to the individual “accuracy” (correct or incorrect) and “response bias” (left or right), which are approximations of sensitivity and criterion, respectively. The results corroborate those of the aggregate data analysis and show that no single subject is driving the effect. Figure 3A plots the amplitude spectrum (amplitude of the vectorial average across subjects, like those in Figure 3C) for oscillations in accuracy, and Figure 3B that for response bias. Response bias (Figure 3B) shows a strong peak around 9.4 Hz, reinforcing the curve-fitting results for criterion in Figure 2C. A similar permutation procedure as for the aggregate analysis yielded the corrected p values plotted in Figures 3D and 3E. For response bias, the oscillation at 9.4 Hz is significant, p = 0.024 (Figure 3F). Although there are several peaks in the amplitude spectrum for accuracy (Figure 3A), none was significant after multiple-comparison correction (Figure 3D).

Figure 3C shows the individual vectors for response bias at 9.4 Hz, with the vector angle showing the phases of individual participants at noise onset. The vectors are tightly clustered around a phase angle of 172° ± 8° SEM and amplitude of 0.023 ± 0.009, similar to the phase angle and amplitude we obtained from the curve-fitting analysis with the aggregate data. In addition, we performed a t test on the average of the individual vectors at 9.4 Hz for the criterion data, evaluating the 2D dispersion of the individual subjects. The result was significant, with t(13) = 2.85 and p = 0.014. Using Equation 8, we obtained a logBF of 0.683, which by convention is considered substantial evidence in favor of the model.

### Oscillations in Response Bias Are Driven by Stimulus History

**Figure 4:**
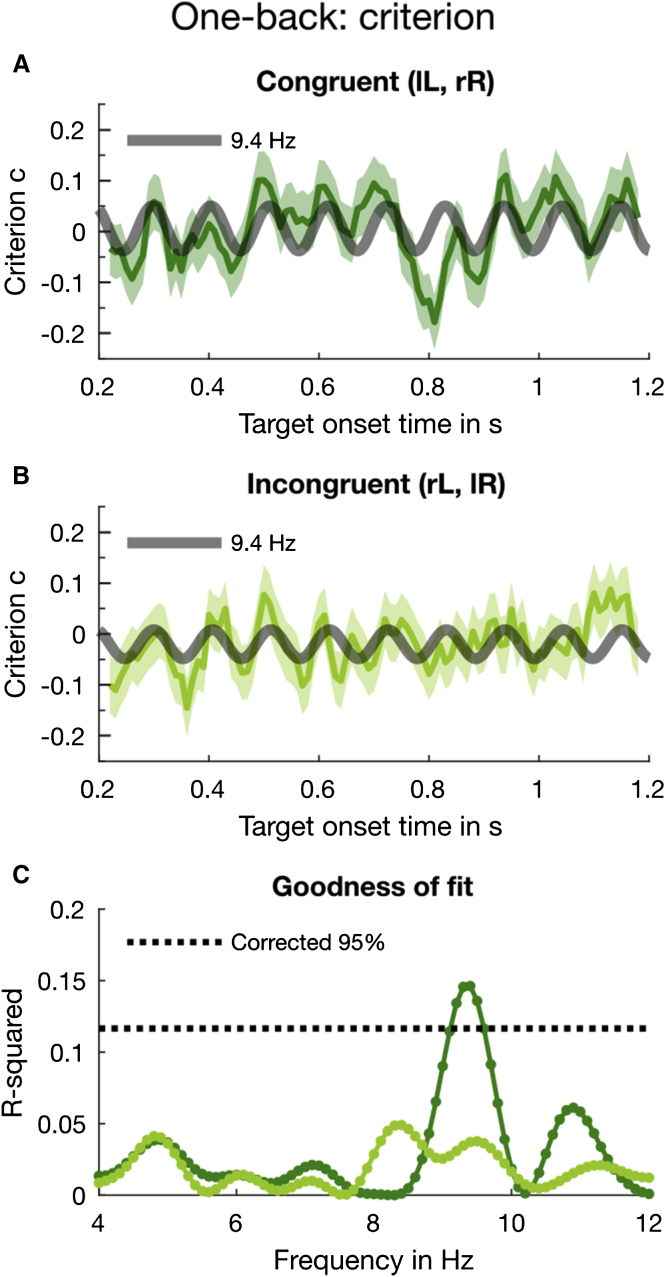
Results of the 1-Back Analysis for Criterion with Aggregate Data Here (and elsewhere) lowercase letters (r or l) refer to the ear of the previous trial, and uppercase (R or L) to the current trial. (A) The dark green line shows the binned *congruent* trials (data smoothed for display purposes only). The error bars indicate ±1 SEM obtained by bootstrapping the aggregate data 2,000 times. The thick gray line represents the 9.4-Hz oscillation, which we fitted to the criterion data. (B) The *incongruent* trials submitted to the same binning, curve-fitting, and bootstrapping procedure as congruent trials. (C) The goodness of fit for congruent (dark green line) and incongruent trials (light green line) at all tested frequencies from 4 to 12 Hz in 0.1-Hz steps. The black dotted line indicates the 95^th^ percentile of the distribution of maximal R^2^ obtained by permuting the individual trials. Table S1 gives an overview of the statistical results. Figure S2 shows the results of the 1-back analysis for sensitivity.

Having established the existence of rhythmic fluctuations in criterion in both the aggregate and individual data, we investigated the dependence of the oscillations on the previous stimulus, using the same two analysis techniques. We separated the trials into two groups, based on whether the previous stimulus had been presented to the same ear (congruent, lL or rR, where L and R denote the ear of origin of the current stimulus and l and r that of the previous stimulus) or different ear (incongruent, lR or rL), and analyzed for criterion. Figure 4 shows the results of the curve-fitting analysis of the aggregate data. Congruent trials (Figure 4A, dark green line) displayed a good fit at 9.4 Hz (thick gray line; R^2^ = 0.15, p = 0.015), with an amplitude of 0.054 ± 0.015 SEM (higher than when all trials were considered). However, the goodness of fit for incongruent trials did not approach significance at any frequency (Figure 4B, light green line; R^2^ = 0.04 and p = 0.9 at 9.4 Hz). Figure 4C shows that only for congruent trials did the goodness of fit survive the multiple-comparison correction, and only for frequencies between 9.1 and 9.6 Hz (p < 0.05, corrected across all tested frequencies). Furthermore, the 2D bootstrap test indicated that the phase (180° ± 19° SEM) and amplitude of this 9.4-Hz oscillation for congruent trials were significant, p = 0.008 (FDR corrected). For incongruent trials, phase and amplitude at 9.4 Hz, 179° ± 26° and 0.029 ± 0.015, respectively, were not significant, p = 0.2, consistent with the sign test on the goodness of fit.

To probe further the relative goodness of fit of the alpha oscillations for the congruent and incongruent trials, we used the Akaike information criterion (AIC [41, 42]) to calculate the relative likelihood of the fit at 9.4 Hz compared with other peaks, separately for the two datasets. For the congruent data, the 9.4-Hz fit was 10.7 times more likely to capture the true model of the data than the next largest peak, at 10.9 Hz. This is strong evidence for a principal modulation at 9.4 Hz describing the data. For the incongruent trials, however, the most probable modulation (at 8.4 Hz) was only 1.4 times more likely than that at the next peak, at 9.4 Hz. The absence of a clear single modulation is consistent with the idea that the incongruent trials are dominated by random noise.

**Figure 5:**
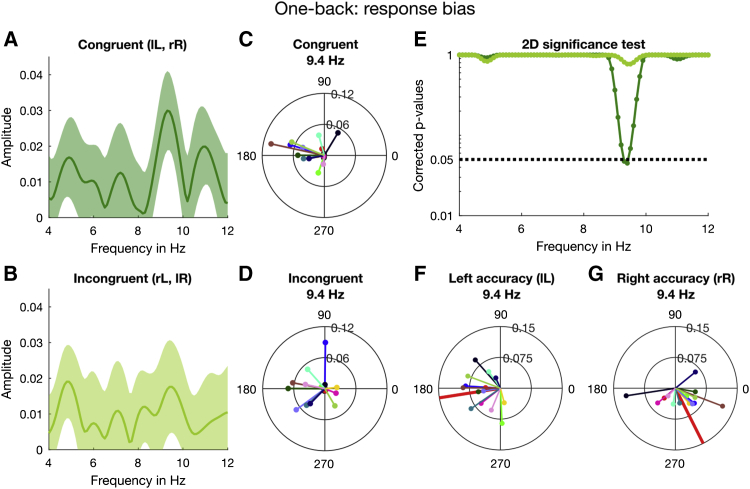
Results of the 1-Back Analysis for Response Bias with Individual Subject Data (A) Amplitude spectrum for the congruent trials computed from the individual estimates of β_1_ and β_2_ averaged across participants. The shaded area around the dark green line indicates ±1 SEM. (B) By the same method, we computed the amplitude spectrum with incongruent trials. (C) Individual phase and amplitude vectors (at noise onset) based on congruent trials at 9.4 Hz. (D) Individual vectors for incongruent trials at 9.4 Hz. (E) The 2D significance test (see Figure 3F) was done for every frequency from 4 to 12 Hz in 0.1-Hz steps. The dark and light green lines depict the corrected p values for congruent and incongruent trials, respectively. The black dotted line indicates α = 0.05 (corrected for multiple comparisons). (F) As a post hoc test, we split the congruent trials further into trials that contained a left or right target and tested their phase relationship using circular statistics. At 9.4 Hz, observers showed very strong phase coherence for both ears. Here, we plot the individual phase and amplitude vectors for congruent trials containing a target in the left ear. The thick red line indicates the direction of the mean vector (with unit length) which is close to 180°. (G) The individual phase and amplitude vectors at 9.4 Hz for congruent trials containing a right target. The direction of the mean vector is ∼301°. We also conducted two 1-back analyses contingent on the previous response; the results are shown in Figures S3 and S4. An overview of the statistical results is provided in Tables S2 and S3.

**Figure 6:**
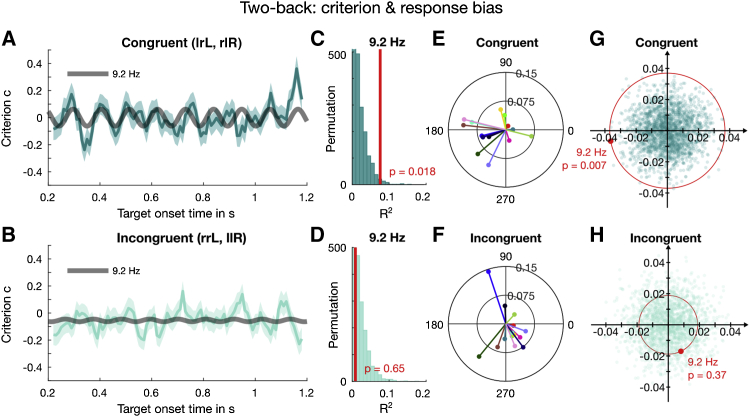
Results of the 2-Back Analysis for Criterion with Aggregate and Individual Data of Trials Incongruent with the Previous Trial and Either Congruent or Incongruent with Stimuli 2 Trials Back (A) The dark cyan line shows the binned congruent trials (error bars indicate ±1 SEM), with the dark gray thick line showing the best fitting sinusoid over the range of 9.1 to 9.6 Hz. (B) The binned incongruent data fitted with the same frequency, 9.2 Hz. (C) The R^2^ obtained at 9.2 Hz (thick red line) was compared against the goodness of fit of the surrogate shuffled data (dark cyan histogram), binned, and fitted as the original data. (D) The same permutation test for the incongruent condition was not significant. (E) The individual vectors in the congruent condition at 9.2 Hz, with subjects color coded as in Figures 3 and 5. Their phases cluster around a similar phase as in the congruent 1-back trial (Figure 5C). (F) The phases of incongruent trials at 9.2 Hz are not consistent across participants (Figures 5C and 5D). (G) The result of the 2D permutation test on the individual subjects’ trials at 9.2 Hz for the congruent condition is significant, consistent with the result of the aggregate data analysis shown in (C). (H) The 2D permutation result for the incongruent condition is not significant, also consistent with the aggregate result in (D). The statistical results are also listed in Tables S1 and S2.

Again, we examined the individual subject data using the regression analysis, separately for congruent and incongruent trials. As for the aggregate data, the congruent trials (dark green line) yielded a large peak around 9.4 Hz with A = 0.03 ± 0.011 (Figure 5A). At this frequency, the amplitude for the incongruent trials is much reduced, with A = 0.019 ± 0.012 (Figure 5B). Inspection of the vector plots shows a tight cluster around a mean phase angle (at noise onset) of 164° ± 8° SEM for congruent trials (Figure 5C) but a greater dispersion for incongruent trials (mean phase 177° ± 10° SEM; Figure 5D), although still not uniformly distributed over 360°. The results of the 2D permutation test plotted in Figure 5E show that the only frequencies to survive the strict multiple-comparison correction were around 9.4 Hz (dark green line, congruent trials) with p = 0.046. In contrast, incongruent trials showed no significant frequencies (light green line; p = 0.8 at 9.4 Hz). Corroborating the 2D permutation results, the t test on the average of the individual vectors at 9.4 Hz was clearly significant for the congruent trials (t = 2.85, p = 0.014, logBF = 1.00). By convention, logBF ≥ 1 is considered strong evidence in favor of the model. The incongruent data did not reach significance (t = 1.53, p = 0.15, logBF = −0.029). However, the logBF near 0 does not allow us to claim with certainty that there were no oscillations at this frequency. Indeed, later analyses (Figure 6) show that there are oscillations for a subset of trials, in agreement also with the non-uniform distribution of the individual phases (Figure 5D).

The analysis of the sensitivity data revealed no significant oscillations at any frequency, either for congruent or incongruent data (Figure S2). Because our previous work suggested that sensitivity may oscillate out of phase in the two ears [20], we performed a post hoc regression analysis of the accuracy of congruent trials separately for ear of origin. Figures 5F and 5G show individual vectors for *both* ears for the *congruent* trials (rR and lL analyzed separately) at 9.4 Hz: left targets led to a mean direction of 190° ± 10° and right targets 301° ± 9°. The Rayleigh test of uniformity [43] indicated that the phase coherence across participants in both the left, z = 6.24, p = 0.001, and right ear was significant, z = 4.47, p = 0.009 (see also Table S3). The mean difference between rR and lL was 111°, broadly consistent with an antiphase relationship. Using the Watson-Williams test (circular analog to a two-sample t test [43]), we further confirmed that the group phase distributions for left- and right-ear accuracy in the congruent trials were significantly different (p < 0.05, Bonferroni corrected) at 9.4 Hz. Thus, the lack of oscillations in the binaural dataset (Figures 2, 3, and S2) may reflect cancellation of out-of-phase left- and right-ear oscillations, consistent with our previous research [20].

An obvious question is whether the oscillations in criteria depend on the current stimulus being the same as the previous stimulus or the previous response. This is difficult to test, as responses were 75% correct, and therefore strongly correlated with stimuli. However, we ran the curve-fitting analysis for current stimuli contingent on the previous response. In this analysis, the main peak remained at 9.4 Hz but was not significant (Figure S3). This suggests—but does not prove—that the trials needed to be coherent with previous stimuli rather than responses. No other analyses, including coherent and incoherent error trials (where responses are different from stimuli), yielded significant results (Figure S4). However, these error analyses necessarily result in greatly reduced numbers of trials (about 25%), making significance difficult to reach.

### Duration of the Serial Effect on Bias

Because the serial-dependence analysis showed strong 2-back effects (Figure 2B), we tested whether measurable oscillations near 9.4 Hz were contingent on stimuli presented two trials back. We examined further the 1-back incongruent data which, although they showed no significant oscillations (Figure 5B), the Bayes factor analysis did not allow us to exclude the possibility that some oscillations exist. We divided trials with incongruent stimuli on the previous trial (i.e., the light green curves in Figures 4B and 5B) into two groups, where stimuli presented two trials back were either congruent (lrL or rlR, where uppercase denotes current trial) or incongruent (rrL or llL). Because we expected oscillations to be weak and near the frequency found for 1-back trials, we confined our analysis window to a limited region of 9.1–9.6 Hz (for both the original and shuffled data). Figure 6A shows a significant oscillation in the 2-back congruent data, best fitted by a sinusoid of ∼9.2 Hz (R^2^ = 0.08, p = 0.018). The amplitude of this oscillation (0.073) was higher than when all incongruent 1-back trials were considered (0.029; see Table S1). There was no modulation at this frequency in the 2-back incongruent data (R^2^ = 0.01, p = 0.7; Figure 6B). Similarly, the 2D bootstrap test confirmed that the amplitude and phase of the 9.2-Hz oscillation were significant for congruent trials (A = 0.07 ± 0.024, ϕ = 209° ± 20°, p = 0.002), but not for incongruent trials (A = 0.02 ± 0.018, ϕ = 348° ± 33°, p = 0.18).

We examined the individual group coherence at 9.2 Hz with the same regression analysis as before for 2-back congruent (Figure 6E) and 2-back incongruent (Figure 6F) trials. The amplitude of the congruent trials is equal to 0.018 and the phases cluster around 190° ± 10°, similar to that of the 1-back congruent data (Figure 5C) and the result obtained from the aggregate data. For the incongruent trials, the amplitude is 0.019 ± 0.018 and the mean phase is 295° ± 9°, which bears no relation to the mean phase of either the 1-back congruent or incongruent condition (Figures 5C and 5D). Figures 6G and 6H show the results of the 2D permutation test at 9.2 Hz, which are consistent with the results of the aggregate data analysis. Very few points (dark cyan dots in Figure 6G) from the permutation distribution (p = 0.007) exceed the group mean vector (thick red dot) in the congruent condition, compared with the incongruent condition, p = 0.37 (Figure 6H). The 2D t test on the individual vectors at 9.2 Hz (Figure 6E) also indicated that the oscillation in the congruent trials was significant, t(13) = 2.13, p = 0.05, logBF = 0.4, but not the oscillation in the incongruent trials, t(13) = 1.07, p = 0.3, logBF *=* −0.29. Taken together, the results suggest that the 9.2-Hz oscillation lasts at least two trials.

To be certain that there was no significant oscillation in the incongruent trials, we compared further the 2-back congruent and incongruent trials, with both a bootstrap analysis of the aggregate data (similar to the one we used in [20]) and a standard t test on the individual vectors. Because stimuli presented either one or two trials back can both generate oscillations, we divided the data into those totally congruent over three trials (llL and rrR) and totally incongruent (rrL and llR). As in the previous analysis (Figure 6), each set comprises a quarter of the total trials.

**Figure 7:**
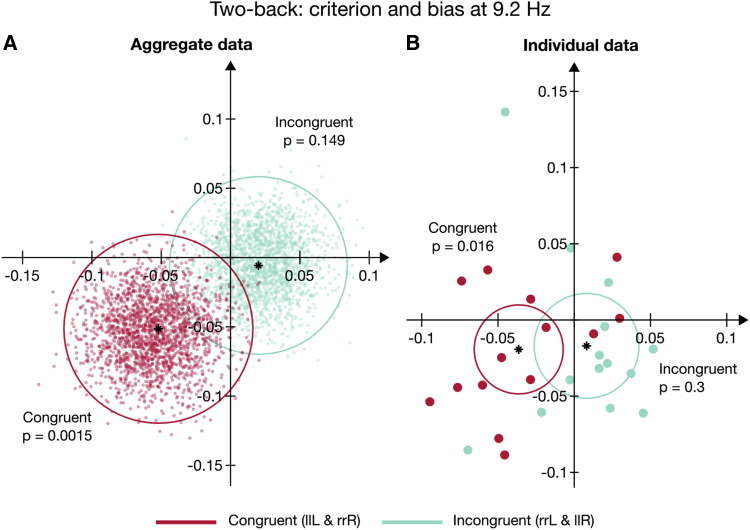
Results of the Aggregate and Individual Subject Data Analyses for Totally Congruent (red, llL and rrR) and Totally Incongruent Trials (cyan, rrL and llR) at 9.2 Hz (A) Bootstraps of the aggregate data obtained by 2,000 random draws, with replacement and the binning and fit procedure as in Figure 2. The red and cyan circles indicate the 95% confidence regions. The thick black asterisks show the vectors of the original data. The p values reflect the proportion of bootstrap samples that belong to the semi-plane opposite the original vectors. This is defined by a line (not shown; but see [20]) passing through the origin and orthogonal to the phase angles of the original data. (B) The red and cyan dots represent the individual vectors and the black asterisks indicate their vector averages at 9.2 Hz for congruent and incongruent trials, respectively. As in (A), the red and cyan circles indicate the 95% confidence regions. The p values were computed using a 2D t test. The curve fit, amplitude spectrum, and individual phases for the totally congruent condition are shown in Figure S5. Tables S1 and S2 provide an overview of the statistical results.

Figure 7A shows the bootstrap results (2,000 independent random draws, with replacement, before binning) of the aggregate data at 9.2 Hz for congruent (red dots) and incongruent (cyan dots) trials. The average aggregate amplitudes for congruent trials (black asterisks), A *=* 0.073 ± 0.024 SEM (see also Figure S5), are much greater than for incongruent trials, A *=* 0.021 ± 0.018 SEM (see also Figure 6B). The 95% confidence regions (red circle) for the congruent condition does not even approach zero, whereas the incongruent samples (the cyan circle) embrace fully the origin. For congruent trials, only three points cross the semi-space opposite the mean vector, leading to a significance of p = 0.003. On the other hand, 18% of the incongruent bootstrapped data lie on the semi-space opposite the average vector, consistent with the oscillation being random.

The vector plot of individual participants in Figure 7B suggests a similar story. The average amplitude across subjects is lower for incongruent, A = 0.019 ± 0.018 SEM, than congruent trials, A = 0.041 ± 0.015 SEM. Furthermore, whereas the mean phase across subjects in the congruent condition, ϕ = 208° ± 10°, is similar to that from the aggregate data, 209° ± 10° SEM (by bootstrap), the mean phase across subjects in the incongruent condition, ϕ = 295° ± 9°, is different from that from the aggregate data, 348° ± 33° SEM (by bootstrap). This is consistent with the idea that the 9.2-Hz bias fluctuation in the incongruent trials is principally noise, given that applying two different methods yields two different results. Finally, we confirmed with the 2D t test that the oscillation in the fully congruent trials was significantly different from zero (t = 2.77, p = 0.016, logBF = 0.93), whereas for the incongruent trials, the difference was not significant (t = 1.07, p = 0.3, logBF = −0.29). The negative log Bayes factor is consistent with there being no modulation other than noise.

## Discussion

To generate and maintain a stable and coherent percept from noisy and ambiguous signals, perceptual systems take advantage of past information to anticipate forthcoming sensory input. Although there is a good deal of behavioral evidence in favor of this predictive account of perception, little is known about the underlying neural mechanisms. The current study suggests that predictive perception is linked to rhythmic alpha-band oscillations. Performance in identifying the ear of origin (and consequently the location in space) of a weak tone was rhythmically biased by previous stimuli presented one or even two trials before the current stimulus. Although the immediately past trials had no *average* serial effect (discussed below), we observed a strong 9.4-Hz rhythmic fluctuation in bias (or criterion), which was critically dependent on stimulus history: perceptual oscillations occurred *only* when a stimulus had previously been presented to the same ear as the current one, either one or two trials back. The strong dependence of oscillations on stimulus congruency suggests that they play a fundamental role in the propagation of predictive information, possibly related to the “perceptual echo” suggested by VanRullen and Macdonald [31].

Although we define stimulus congruency in terms of ear of origin, it is important to note that stimuli confined to one ear are perceived as originating from that side of space. Therefore, the interaction between consecutive stimuli may be mediated by the neuronal circuitry defining acoustic space, rather than within the monaural circuitry (future research may use external speakers rather than headphones). Oscillation in the representation of space mechanisms would be consistent with several studies in vision reporting theta-band behavioral oscillations between spatial locations [44] and between objects at different spatial positions [45]. Interestingly, all these studies showed that positions in left and right hemispace induce oscillations in performance that are out of phase with each other. Similarly, Lozano-Soldevilla and VanRullen [46] report opposite EEG phases in the two hemispheres of 10-Hz reverberations in response to visual stimulation (perceptual echoes). However, a number of studies have failed to observe similar echo phenomena in audition [24, 33]. The contradictory findings between vision and audition may be due to the general difficulty in measuring alpha oscillations in audition using non-invasive EEG, whereas recent intracranial recordings point unequivocally to the existence of auditory alpha with similar properties to that in vision [47]. Furthermore, most auditory studies use *diotic* stimulation contributing to the conflicting results [22, 24, 33, 48]. Considering the antiphase relationship between the left- and right-ear oscillations in performance (proportion correct, or sensitivity) observed here and in our previous study [20], these may lead to a cancellation of oscillatory effects reported previously.

The major result of this study is that oscillations in bias are dependent on stimulus congruency. “Serial dependence,” the tendency of judgments of many perceptual attributes to be biased toward previously presented stimuli [3, 4], is an important signature of predictive perception. Under the assumption that the world tends to remain stable, the previous stimulus acts as a Bayesian prior, optimizing performance when appropriately combined with the current stimulus [34]. We presume a similar process occurs here. At the onset of each trial (initiated by a binaural noise burst), observers begin to seek the ear of origin (or spatial position) of the target tone within the noise, considering both the sensory evidence of the new stimulus and past perceptual history. If the stimulus had not been presented to a particular ear on the previous two trials, there will be no prior within that circuitry, so only the current stimulus is to be considered. On the other hand, if a stimulus had previously been presented to that ear, it should influence the response to stimuli to that ear.

Crucially, our data show that the combination of the sensory signal with the congruent prior leads to perceptual oscillations in the alpha band. Several possible mechanisms may generate the oscillatory behavior, but two are most consistent with the current literature. The memory trace itself may oscillate at alpha frequencies, as has been recently shown by Huang et al. [30] for visual memory. Alternatively, the start of each trial may generate a loop of reverberating signals between the top-down memory signals and the bottom-up sensory signals, with the delay of the reverberation generating an excitatory/inhibitory modulation of the sensory response. With either explanation, the oscillations should be synchronized to the onset of each trial, signaled by the onset of binaural noise. Because each ear conveys a different signal, the oscillations should be out of phase.

It may seem counterintuitive that oscillations in criterion should be confined to the ear that had received the prior signal. Indeed, this fact constrains the interpretations of the data. If the oscillations occurred at the decision level, we would expect both ears to be affected, as the criteria oscillated between one ear and the other. That the oscillations are confined to the ear where the previous stimuli were presented suggests that the interaction is not at the decision level but within the sensory circuits themselves (either the monaural circuitry or that defining spatial location). This is in line with evidence that serial dependence works on perceptual processes, rather than at decision or response stages [35] (although there is some controversy on this point [49]). It is also consistent with the literature on working memory showing that the regions of the brain involved in sensory processing of a given perceptual attribute are modulated by working memory specific for that attribute (for a review, see Pasternak and Greenlee [50]). In match-to-sample tasks, the interactions between working memory and stimuli are highly sensory specific [50]. In particular, Gottlieb et al. [51] have reported context-dependent potentiation of neural activity in the monkey auditory cortex. In a match-to-sample auditory task, the neural response to a specific tone was enhanced after presentation of a sample of matched frequency. They suggest that the sample changes the synaptic neural efficacy transiently, enhancing the response to the test. A similar mechanism could be at work in our study. The previous tones alter the synaptic efficiency on specific circuits, so a target to the same ear (space) is amplified whereas targets to the other ear are unaffected. As discussed above, the modulation is rhythmic, pointing to reverberating mechanisms.

If the behavioral oscillations result from rhythmical variation of neuronal sensitivity (rather than in decision boundary), why should they affect criterion rather than sensitivity? First, because the target ear was chosen randomly on each trial, the previous trial was uninformative about the ear of origin of the signal, and could therefore not on average affect sensitivity, only bias or criterion. Second, both this and our previous [20] study suggest that the oscillations may have an opposite starting phase in each ear. Because d′ can be calculated only after a combination of hits and false alarms of both ears, the out-of-phase modulations should cancel each other out. Post hoc tests of congruent trials separated for ear of origin of the signal (Figures 5F and 5G) are consistent with this suggestion. On the other hand, combining the counter-phased modulation for criterion (the sum of hits and false alarms, oppositely signed for each ear) will sum the modulations in the binaural measurements. In this interpretation, modulation of criterion is associated with perceptual changes [52, 53], rather than with modulation of a decision boundary.

If the role of serial dependence is to bias perception toward recent perceptual history (on the assumption of perceptual continuity), we should expect a positive serial dependence (averaged over all delays) on the immediately previous trial (1-back), as is normally observed in studies of serial dependence [3, 4, 5, 6, 7, 8, 9, 10]. That we found robust 2-back effects strongly suggests that 1-back effects were also present, but not revealed in our data. We propose two possible (non-mutually exclusive) explanations of why this may be so. One is that forced-choice paradigms can lead to sequential response biases, such as alternation [37], which would tend to cancel out positive serial dependence based on the previous stimulus (usually measured by reproduction [3, 4]). That the response-based analysis showed negative serial dependency is consistent with response alternation, which would have cancelled perceptual positive serial dependence. The clear average serial dependence for trials two back is also consistent, as double alternation of response would not lead to cancelation. Another possibility is that the stimuli may have caused both positive serial dependence and negative adaptation aftereffects. Negative aftereffects tend to be shorter lived than assimilative dependencies, and may therefore cancel out only 1-back, not 2-back, trials [11]. Whatever the reason for the lack of positive serial dependence in the averaged 1-back results, our study shows that oscillations may be a more sensitive signature of memory-based perceptual effects than simply looking at average results. Many competing effects could reduce or annul the measurement of average serial-dependence effects, without affecting rhythmic, time-dependent oscillations.

To summarize, we have shown that when discriminating the ear of origin of a brief monaural pure tone within dichotic white noise, responses are biased rhythmically through alpha oscillations when the previous target had been presented to the same ear. To account for these findings, we propose that presentation of a target potentiates circuit-specific reverberations that rhythmically bias perceptual performance. The exact mechanisms of this process are yet to be understood, but it is clear that alpha rhythms play a major role in combining expectations and past perceptual history with sensory signals. It would be interesting to study these effects further with neurophysiological techniques, such as EEG, magnetoencephalography (MEG), or functional near-infrared imaging.

## STAR★Methods

### Key Resources Table

**Table:** 

REAGENT or RESOURCE	SOURCE	IDENTIFIER
**Deposited Data**		

Source data for figures	This study	https://doi.org/10.17605/OSF.IO/SWQ4N

**Software and Algorithms**		

MATLAB 2018b	The MathWorks	RRID: SCR_001622
Psychophysics Toolbox 3	[49]	RRID: SCR_002881

**Other**		

DATAPixx	Vpixx Technologies	RRID: SCR_009648
ResponsePixx	Vpixx Technologies	N/A
Curve Fitting Toolbox	The MathWorks	N/A
ER-2 in-ear tube phones	Etymotic Research	N/A

### Lead Contact and Materials Availability

Further information and requests for resources should be directed to and will be fulfilled by the Lead Contact, David Burr (dave@in.cnr.it). There is no restriction for distribution of materials.

### Experimental Model and Subject Details

Eighteen healthy adults with normal hearing took part in the experiment. Three were excluded for imbalanced left and right ear auditory thresholds and one for very long reaction times (2.5 standard deviations from the group mean). Of the remaining 14 participants (mean age 21.14 ± 4.22), 4 were male and 2 left-handed. All participants provided written, informed consent. The study was approved by the Human Research Ethics Committees of the University of Sydney. We based our sample size estimations on our previous study [20], which showed oscillations in auditory perceptual performance, and other studies on similar behavioral rhythms in vision [44, 54], without running a formal power analysis.

### Method Details

Participants sat in a dark room and listened to auditory stimuli via in-ear tube-phones (ER-2, Etymotic Research, Elk Grove, Illinois) with earmuffs (3M Peltor 30 dBA) to isolate external noise. On each trial, 2 s of dichotic broadband white-noise (randomly generated each trial and uncorrelated between the two ears) were presented together with a monaural target tone. The noise burst served to reset potential oscillations, similar to a visual or auditory cue [44, 45, 54] and action or saccadic execution [39, 55, 56, 57]. The target (1000 Hz, 10 ms) was delivered randomly with equal probability to either ear during the 2 s noise burst, within 0.2–1.2 s from noise onset. For each ear, the target intensity was kept near individual thresholds (75% accuracy), using an accelerated stochastic approximation staircase procedure [58, 59]. Participants reported the *ear of origin* of the tone via button press (ResponsePixx, Vpixx Technologies, Saint-Bruno, Quebec). The next trial started after a silent inter-trial interval (ITI) of random duration ranging from 1.2–2.2 s. Participants completed 2,800 trials (40 blocks of 70 trials with rests between blocks, and no feedback) after a practice block of 20 trials with feedback. Stimuli were presented using the software *PsychToolbox* [60] in conjunction with *DataPixx* (Vpixx Technologies) in MATLAB (Mathworks, Natick, Massachusetts). Trials were excluded if the response occurred before the target onset or after the noise offset, or if the reaction time (RT) exceeded the 99% confidence interval of that individuals’ RTs. In addition, we discarded trials where the target intensity exceeded the 95% confidence interval of individuals’ thresholds.

### Quantification and Statistical Analysis

#### Signal detection theory

To separate sensitivity and response bias, we computed d-prime, *d’*, and the decision criterion, *c*, using Equations 1 and 2 from *signal detection theory* (SDT) [61, 62]. As illustrated in Figure 1B, the calculation of the hit rate (H_*right*_) was based on the hits from the right target condition and the false alarm rate (FA_*left*_) based on the false alarms from the left target condition. *d’* is given by the difference between z-transformed hit and false alarm rates:

(Equation 1) $$d^{'}=\frac{z\left(H_{right}\right)-z\left(FA_{left}\right)}{\sqrt{2}}.$$

The bias of the responses was defined as positive for the left ear:

(Equation 2) $$c=-0.5\times \left(z\left(H_{right}\right)+z\left(FA_{left}\right)\right).$$

#### Aggregate data analysis

We performed two analyses, one based on aggregate data (allowing sufficient data to bin trials into fine time-bins for analysis of sensitivity and criterion), the other on individual data, allowing us to see variability across subjects. For the aggregate data analysis, trials were pooled across all 14 participants and grouped into one hundred 10-ms bins, from 0.2 to 1.2 s post noise onset. The mean number of trials per bin was 151 ± 24 for the left-target condition and 152 ± 25 for the right-target condition. For each bin, we computed *d*’ and *c* as above and fitted the time-series with a sinusoidal function given by:

(Equation 3) $$f\left(t\right)=A\;\cos\left(2\text{πf}t+\phi \right)+a_{0},$$

where *t* is time, *a*_*0*_ a constant and *A* and ϕ the amplitude and phase of the sinusoidal fit, all free parameters. The frequency parameter *f* was fixed between 4 and 12 Hz in 0.1 Hz steps and a non-linear least-squares method was used to obtain the best fit for each tested frequency (a standard implementation in MATLAB with 400 iterations in total). Sensitivity displayed a decreasing non-linear trend over time (see also [20]), which we removed before curve fitting. Detrending was not required for the criterion time series. The goodness of fit *R*^*2*^ was used to test the significance of every fit by applying a permutation procedure [63]: responses of each individual trial were randomized over all SOAs to generate 2,000 surrogate datasets, which we submitted to the same binning and curve fitting procedure as the original data. To correct for multiple comparisons, we determined the maximal *R*^*2*^ for every surrogate dataset irrespective of frequency. This resulted in a distribution of 2,000 maximal *R*^*2*^ (Figures 2D and 2E), against which we compared each fit to the original dataset. Any frequency that exceeded the 95 percentile of the maximal-*R*^*2*^ distribution (dotted line in Figure 2C) was considered significant. We also estimated the variability in the original aggregate data by applying the bootstrap method. We randomly selected the same number of trials (with replacement) from the original data 2,000 times, and each surrogate dataset was submitted to the same binning and curve fitting procedure as above.

#### Akaike information criterion

To estimate the relative quality of the harmonic model of Equation 3, we computed the *Akaike information criterion* (AIC) using the *residual sum of squares* (RSS) with n = 100 (number of data bins) and *k* = 2 (number of parameters):

(Equation 4) AIC=2k+nlnRSS.

#### Individual and group analyses

To examine the individual data for oscillations and evaluate their coherence across subjects, we used an approach based on single trials (for similar approaches, see [32, 39]). The response *y*_i_ (*i* = 1, 2… *n*, where *n* is the total number of trials) to a target at time *t*_i_ (i.e., the interval from noise onset to target onset in seconds) is modeled by the linear combination of harmonics at each tested (angular) frequency as follows:

(Equation 5) $${\hat{Y}}_{n}=\beta_{0}+\beta_{1}\;\sin\left(2\text{πf}t_{n}\right)+\beta_{2}\;\cos\left(2\text{πf}t_{n}\right),$$

where *Ŷ*_*n*_ represents the predicted responses and β_0_, β_1_ and β_2_ are fixed-effect regression parameters estimated using the *linear* least-squares method of MATLAB (*fitlm* function from the *Statistics and Machine Learning* toolbox). This *general linear model* (GLM) model estimates the regression parameters adequately when the sampling rate is uniform across the time series. As this condition may not always be met at the individual subject level, we included a third independent regressor containing information about the stimulus:

(Equation 6) $${\hat{Y}}_{n}=\beta_{0}+\beta_{1}\;\sin\left(2\text{πf}t_{n}\right)+\beta_{2}\;\cos\left(2\text{πf}t_{n}\right))+\beta_{3}\text{S}\left(t_{n}\right),$$

where *S* is the stimulus at time *t* and takes the value –1 or +1 for left and right target, respectively. For sensitivity, *y*_i_ = 1 for correct and *y*_i_ = −1 for incorrect responses, and for response bias, *y*_i_ = 1 for a ‘right’ response and −1 for ‘left’. Although binary responses can be modeled with a logit or probit link function, non-linear transformation of *Ŷ*_*n*_ made no difference in our study (for further discussion, see [64]), so we performed no transformation of *Ŷ*_*n*_. Using Monte Carlo simulation, we tested that the present GLM implementation derives the exact amplitude and phase of a sinusoidal function, also in presence of high noise.

The significance of the model fit in Equation 6 was evaluated with a two-dimensional permutation test: we shuffled the SOAs of each individual’s trials to create 2,000 surrogate datasets per subject and fitted each dataset with the model described in Equation 6. As with the original data, the resulting β_1_ and β_2_ were averaged across subjects for every frequency tested. This yielded a joined distribution of 2,000 surrogate means for each frequency from 4-12 Hz in 0.1-Hz steps. To correct for *multiple comparisons*, we determined the *maximal vector* of each joined distribution irrespective of the frequency. This resulted in a joint distribution of 2,000 maximal vectors, against which we compared the original group mean.

To evaluate the error in amplitude and phase across participants, we computed the average 2D scatter of the individual data points (Figure 7B) using:

(Equation 7) $$SD=\sqrt{\frac{{\left(\sum \left|\vec{v}-m\vec{v}\right|\right)}^{2}}{N}},$$

where *N* is the number of individual vectors. We obtained the amplitude SEM by projecting the SD values along the average vector ($m\vec{v}$*)* and divided them by the $\sqrt{N-1}$. A similar procedure was applied to calculate the phase SEM, using propagation of errors. We then obtained the one-sample 2D *t*-statistic from the ratio of the amplitude of the mean vector to the SEM of the amplitude. We computed the Bayes factors for this 2D t test by applying the Bayesian Information Criterion (BIC) approximation from [65], implemented in the freely available MATLAB toolbox *BayesFactor* [66], using the one-sample *t*-statistic for amplitude:

(Equation 8) $$BF_{10}\approx \frac{1}{\sqrt{n{\left(1+\frac{t^{2}}{df_{2}}\right)}^{-n}}},$$

where *n* is the sample size (14 participants) and *df*_*2*_ is the degree of freedom associated with the error term.

### Data and Code Availability

For data analysis, we used off-the-shelf routines available in MATLAB (version R2018b) in combination with the MATLAB *Curve Fitting* and *Statistics and Machine Learning* toolbox. Source data for the figures in the paper are available (https://doi.org/10.17605/OSF.IO/SWQ4N).

## Supplemental Information

Document S1. Figures S1–S5 and Tables S1–S3

Document S2. Article plus Supplemental Information
