# Supplementary material for: Auditory Perceptual History Is Propagated through Alpha Oscillations
Source: Curr Biol. 2019 Dec 16;29(24):4208–4217.e3. doi: 10.1016/j.cub.2019.10.041 (PMC6926473; doi:10.1016/j.cub.2019.10.041)
Supplement: Document S1. Figures S1–S5 and Tables S1–S3 [file mmc1.pdf]

Current Biology, Volume *29*

Supplemental Information

Auditory Perceptual History Is Propagated through Alpha Oscillations

Hao Tam Ho, David C. Burr, David Alais, and Maria Concetta Morrone

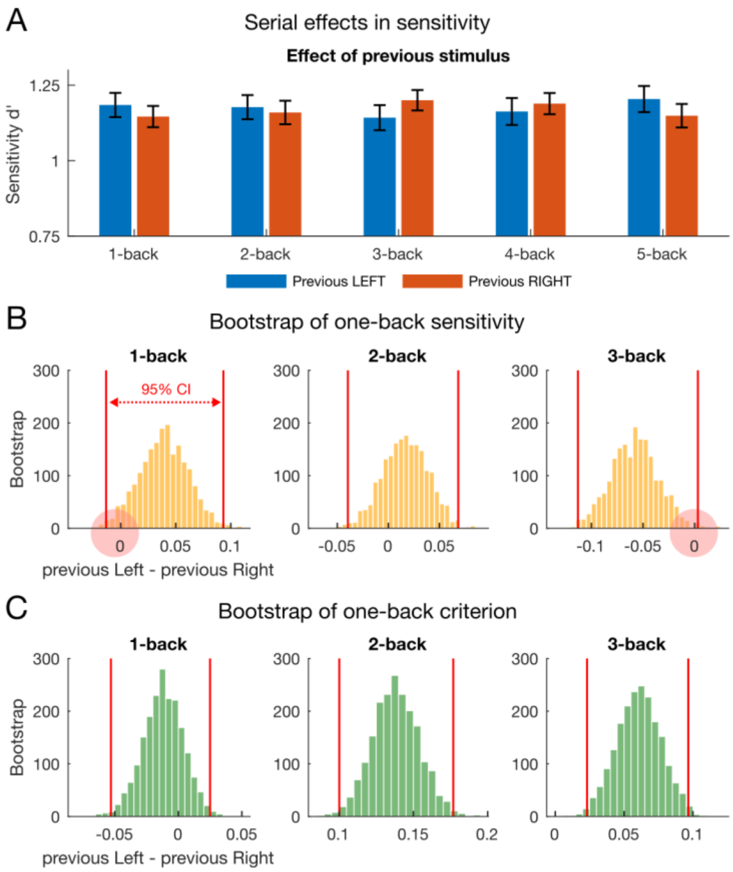

Figure S1. Serial effects in sensitivity and bootstrap results. Related to Figure 1. (**A**) Results of the overall serial-dependence analysis for sensitivity. Group mean sensitivity (*d'*, Eq. 1) computed contingent on the ear of origin of the preceding 1 to 5 targets. Blue and red bars represent previous left and previous right stimulus, respectively. Except for the 3-back (p = 0.01, *logBF* = 1.32), stimulus history had no significant effect on sensitivity after Bonferroni correction. For the 1-back, the paired-sample *t*-test yielded only a marginally significant difference between the contingent left and right, p = 0.06 (corrected), *logBF* = 0.67. (**B**) To evaluate the robustness of the serial effects in sensitivity for 1- to 3-back, we bootstrapped (randomisation with replacement) the individual subject responses 2,000 times, computed the *d'* contingent on the previous left and right stimulus and subtracted the respective group averages. This yielded a distribution of previous-Left – previous-Right differences (yellow histograms), based on which we calculated the 95% confidence intervals (CI), corrected for multiple comparisons (α = 0.05/3). For sensitivity, the CIs (red vertical lines) all included zero, suggesting that we *cannot* safely reject the null hypothesis that the previous left and right stimulus had no different effect on the current trial sensitivity, even if the Bayes factors imply that a difference is more likely than no difference. (**C**) For comparison, we ran the same bootstrap test on criterion for 1- to 3-back. The effect of stimulus history on observers' decision criteria in the 2- and 3-back is clearly robust: the corrected 95% CIs (red vertical lines) do not include zero, corroborating the *t*-test results (see *Averaged effects of stimulus history* in *Results*).

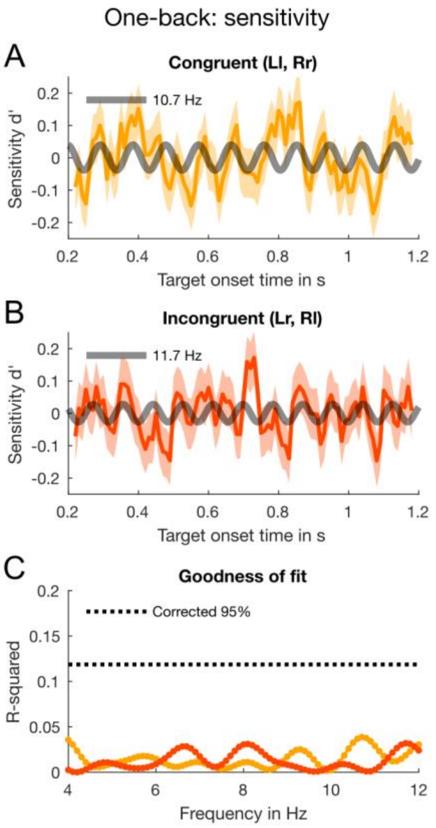

Figure S2. Results of the one-back analysis for sensitivity with aggregate data. Related to Figure 4. Although there was no significant oscillation in sensitivity when all trials were considered, we still evaluated the sensitivity fluctuations in congruent and incongruent trials for comprehensiveness. **(A)** The yellow line shows the binned *congruent* trials (data smoothed for display purposes only). The error bars indicate ±1 SEM obtained by bootstrapping the aggregate data 2,000 times. The thick grey line shows the best-fitting sine function of 10.7-Hz. This fit was not significant, as the results in (C) show. **(B)** The *incongruent* trials were submitted to the same binning, curve fitting and bootstrapping procedure as the congruent trials. The best fit (grey line) was at 11.7 Hz. Again, this fit was not significant. **(C)** The goodness-of-fit for congruent (orange line) and incongruent trials (yellow line) at all tested frequencies from 4-12 Hz in 0.1-Hz steps. The black dotted line indicates the 95 percentile of the distribution of maximal R_2 obtained by permuting (i.e., randomisation without replacement) the individual trials. None of the tested frequencies approached the 95% confidence threshold.

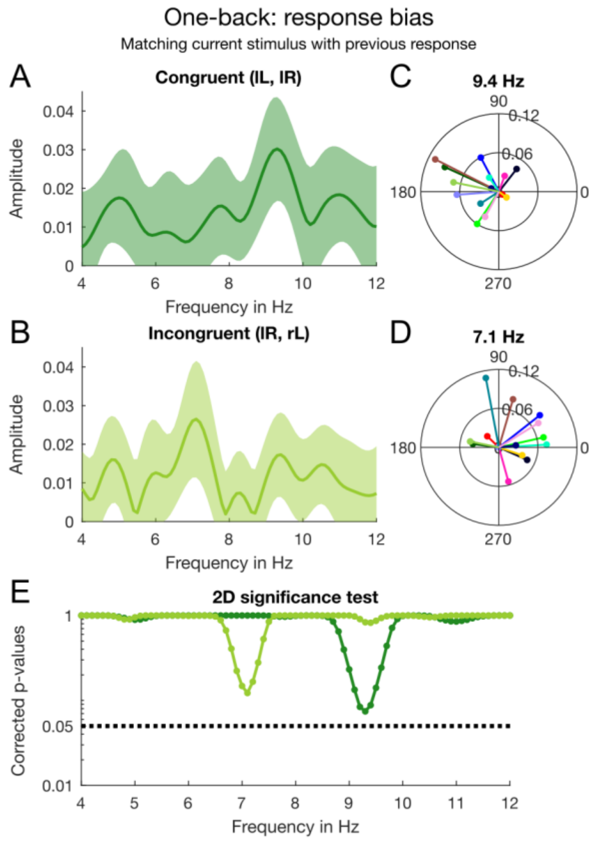

Figure S3. Results of the one-back analysis for response bias with individual subject data contingent on whether the *current stimulus* matches the *previous response*. Related to Figure 5. (A) Amplitude spectrum for the congruent trials on which the current stimulus matches the previous response, computed from the individual estimates of β_1 and β_2 averaged across participants. The shaded area around the dark green line indicates ±1 SEM. As the threshold was set to 75% accuracy, it is possible that the peak around 9.4 Hz is mainly due to correct trials (Figure S4 shows the results for only the incorrect trials). **(B)** By the same method, we computed the amplitude spectrum for incongruent trials where the current stimulus *does not match* the previous response. The peak in amplitude around 7.1 Hz is not significant, as shown in (E). **(C)** Individual phase and amplitude vectors (at noise onset) based on congruent trials at 9.4 Hz. **(D)** Individual vectors for incongruent trials at the peak frequency, 7.1 Hz. **(E)** Using the 2D significance test (see Figure 3F), we evaluated every frequency from 4-12 Hz in 0.1-Hz steps. The dark and light green lines depict the corrected *p*-values for congruent and incongruent trials, respectively. The black dotted line indicates $\alpha = 0.05$ (corrected for multiple comparisons). None of the peaks in (A) or (B) was significant.

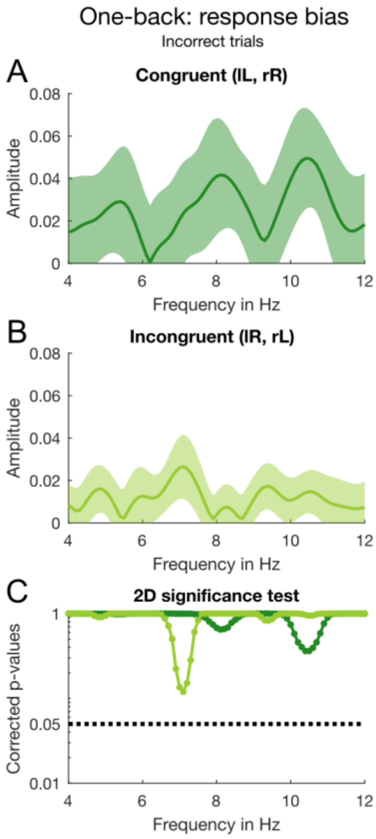

Figure S4. Results of the one-back analysis for response bias with individual subject data contingent on whether the *current stimulus* matches the *previous response*. Related to Figure 5. This is a subset of the data shown in Figure S3 containing only *incorrect* trials. **(A)** Amplitude spectrum for the congruent trials (where the current stimulus matches the previous response) computed from the individual estimates of β_1 and β_2 averaged across participants. The shaded area around the dark green line indicates ±1 SEM. **(B)** By the same method, we computed the amplitude spectrum with incongruent trials (where the current stimulus *does not match* the previous response). **(C)** Individual phase and amplitude vectors (at noise onset) based on congruent trials at 9.4 Hz. **(D)** Individual vectors for incongruent trials at 9.4 Hz. **(E)** The 2D significance test (see Figure 3F) was done for every frequency from 4-12 Hz in 0.1-Hz steps. The dark and light green lines depict the corrected *p*-values for congruent and incongruent trials, respectively. The black dotted line indicates $\alpha = 0.05$ (corrected for multiple comparisons). None of the peaks in (A) or (B) was significant.

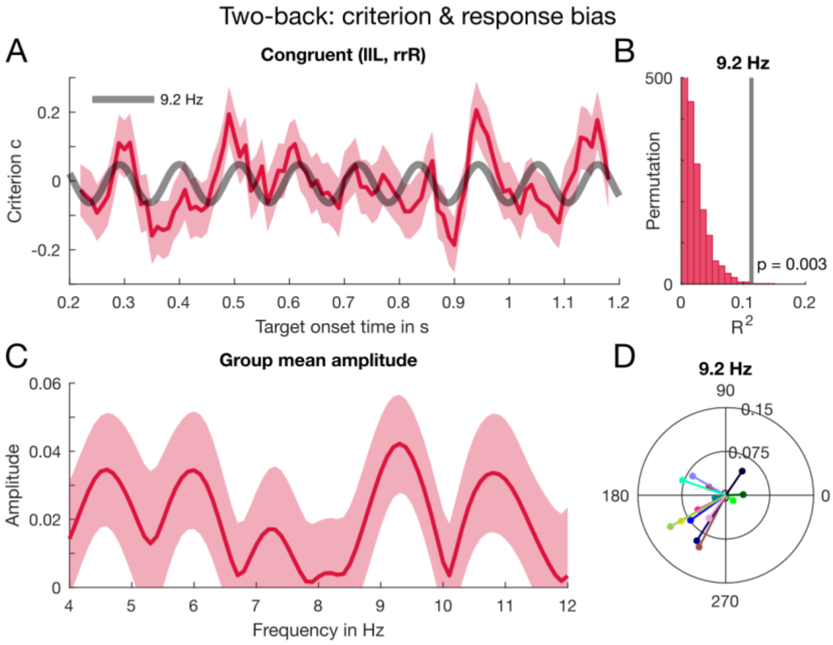

Figure S5. Results of the two-back analysis for criterion and response bias with aggregate and individual data trials fully congruent with stimuli 2 trials back. Related to Figure 7. (A) The red line shows the *binned* data, and the shaded area around the line indicates ±1 SEM (by bootstrap). The thick grey line represents the best fitting sinusoid (9.2 Hz, R_2 = 0.11) over the range of 9.1 to 9.6 Hz. **(B)** The R_2 at 9.2 Hz (thick red line) was compared against the goodness-of-fit of the surrogate shuffled data (red histogram obtained by randomising the aggregate responses without replacement), binned and fitted as the original data. **(C)** The amplitude spectrum computed from the individual estimates of β_1 and β_2 averaged across participants (N = 14). The shaded area around the red line indicates ±1 SEM. **(D)** The individual vectors in the congruent condition at 9.2 Hz, with subjects colour-coded as in Figures 4&6. Their phases cluster around a similar mean phase as in the congruent 1-back (see Figure 5C).

Bias: aggregate data analysis (with curve fitting)

Analysis	Condition	Peak Frequency (in Hz)	Amplitude		Phase (in degree)		Goodness of fit			2D bootstrap sign test	
			A	*SE*	ϕ	*SE*	*R-squared*	*p*		*p*	
All trials		9.4	0.039	0.011	179	18	0.15	0.006	Max corrected	0.010	FDR corrected
1-back	Congruent	9.4	0.054	0.015	180	19	0.15	0.015		0.008	
	Incongruent	9.4	0.029	0.015	179	26	0.04	0.919		0.197	
2-back	c2i1	9.2	0.073	0.024	209	20	0.08	0.018	Post-hoc	0.002	Post-hoc
	c2c1	9.2	0.073	0.024	225	20	0.11	0.003		0.003	
	i2i1	9.2	0.021	0.018	348	33	0.01	0.650		0.182	

Table S1. Results of the criterion analysis with aggregate data (pooled across 14 subjects). Related to Figures 2, 4, 6-7 and S5. The table shows the aggregate amplitude (*A*) and phase (ϕ) of the best-fitting sine curve (peak frequency). SE indicates ± 1 standard error (obtained by bootstrap). The fit was evaluated by comparing the goodness of fit (R_2) of the original data against the maximal R_2 obtained by permutation (i.e., randomising the responses, without replacement), irrespective of frequency (4-12 Hz in 0.1-Hz steps). In addition, we ran a 2-dimensional (2D) sign test on the bootstrapped data (randomisation with replacement) and corrected for multiple comparisons using FDR. See STAR Methods for details. *c2i1:* congruent 2-back, incongruent 1-back; *c2c1:* fully congruent, i.e., both congruent 1- and 2-back; *i2i1:* fully incongruent, i.e., both incongruent 1- and 2-back with respect to the current trial.

Bias: group analysis (with linear regression on individual single trials)													
Analysis	Condition	Peak Frequency (in Hz)	Amplitude		Phase (in degree)		2D sign test		2D t-test (Post-hoc)			Rayleigh test (Phase only)	
			M	*SE*	*M*	*SE*	*p*		*t*	*p*	*logBF*	*z*	*p*
All trials		9.4	0.023	0.009	172	8	0.024	Max	2.48	0.028	0.683	2.67	0.067
1-back	Congruent	9.4	0.030	0.011	164	8	0.046	corrected	2.85	0.014	1.002	5.64	0.002
	Incongruent	9.4	0.019	0.012	177	10	0.776		1.53	0.149	-0.029	1.17	0.315
2-back	c2i1	9.2	0.037	0.017	190	10	0.007	Post-hoc	2.13	0.053	0.401	1.47	0.233
	c2c1	9.2	0.041	0.015	208	10	0.003		2.77	0.016	0.928	3.63	0.024
	i2i1	9.2	0.019	0.018	295	9	0.372		1.07	0.303	-0.294	3.63	0.024

Table S2. Results of the response bias analysis with individual data. Related to Figures 3, 5, 6-7 and S5. The table shows the amplitude and phase of the vector means (*M*) at the peak frequency. SE indicates ± 1 standard error of the mean. The fit was evaluated by comparing the mean vector of the original data against the maximal mean vectors obtained by permutation (i.e., randomising the responses, without replacement), irrespective of frequency (4-12 Hz in 0.1-Hz steps). In addition, we ran a post-hoc 2-dimensional paired-sample *t*-test and used the *t*-statistic to compute the Bayes factor (in *log10*), an approximation of the Bayesian Information Criterion (BIC). See STAR Methods for details. *c2i1:* congruent 2-back, incongruent 1-back; *c2c1:* fully congruent, i.e., both congruent 1- and 2-back; *i2i1:* fully incongruent, i.e., both incongruent 1- and 2-back with respect to the current trial.

Group analysis: congruent left- and right-ear *1-back* sensitivity													
Analysis	Condition	Peak Frequency	Amplitude		Phase (in degree)		2D sign test	2D t-test (Post-hoc)			Rayleigh test (Phase only)	
		(in Hz)	*M*	*SE*	*M*	*SE*	*p*	*t*	*p*	*logBF*	*z*	*p*
1-back	Congruent Right	9.4	0.089	0.029	190	10	0.001	3.17	0.007	1.002	6.24	0.001
	Congruent Left	9.4	0.056	0.032	301	9	0.085	1.31	0.099	0.117	4.47	0.009

Table S3. Results of the sensitivity analysis for congruent *1-back* left and right ear with individual data. Related to Figure 5F&G. The table shows the amplitude and phase of the vector means (*M*) at 9.4 Hz. SE indicates ± 1 standard error of the mean. The fit was evaluated by comparing the mean vector of the original data against the maximal mean vectors obtained by permutation (i.e., randomising the responses, without replacement), irrespective of frequency (4-12 Hz in 0.1-Hz steps). In addition, we ran a post-hoc 2-dimensional paired-sample *t*-test and used the *t*-statistic to compute the Bayes factor (in *log10*), an approximation of the Bayesian Information Criterion (BIC). Finally, we also evaluated the phase coherence (without considering the amplitude) across participants using Rayleigh test of uniformity. See STAR Methods for details. *c2i1:* congruent 2-back, incongruent 1-back; *c2c1:* fully congruent, i.e., both congruent 1- and 2-back; *i2i1:* fully incongruent, i.e., both incongruent 1- and 2-back with respect to the current trial.
